# Lnc2Cancer v2.0: updated database of experimentally supported long non-coding RNAs in human cancers

**DOI:** 10.1093/nar/gky1096

**Published:** 2018-11-08

**Authors:** Yue Gao, Peng Wang, Yanxia Wang, Xueyan Ma, Hui Zhi, Dianshuang Zhou, Xin Li, Ying Fang, Weitao Shen, Yingqi Xu, Shipeng Shang, Lihua Wang, Li Wang, Shangwei Ning, Xia Li

**Affiliations:** 1 College of Bioinformatics Science and Technology, Harbin Medical University, Harbin 150081, China; 2 Department of Neurology, The Second Affiliated Hospital, Harbin Medical University, Harbin 150081, China

## Abstract

Lnc2Cancer 2.0 (http://www.bio-bigdata.net/lnc2cancer) is an updated database that provides comprehensive experimentally supported associations between lncRNAs and human cancers. In Lnc2Cancer 2.0, we have updated the database with more data and several new features, including (i) exceeding a 4-fold increase over the previous version, recruiting 4989 lncRNA-cancer associations between 1614 lncRNAs and 165 cancer subtypes. (ii) newly adding about 800 experimentally supported circulating, drug-resistant and prognostic-related lncRNAs in various cancers. (iii) appending the regulatory mechanism of lncRNA in cancer, including microRNA (miRNA), transcription factor (TF), variant and methylation regulation. (iv) increasing more than 70 high-throughput experiments (microarray and next-generation sequencing) of lncRNAs in cancers. (v) Scoring the associations between lncRNA and cancer to evaluate the correlations. (vi) updating the annotation information of lncRNAs (version 28) and containing more detailed descriptions for lncRNAs and cancers. Moreover, a newly designed, user-friendly interface was also developed to provide a convenient platform for users. In particular, the functions of browsing data by cancer primary organ, biomarker type and regulatory mechanism, advanced search following several features and filtering the data by LncRNA-Cancer score were enhanced. Lnc2Cancer 2.0 will be a useful resource platform for further understanding the associations between lncRNA and human cancer.

## INTRODUCTION

Cancers are a leading cause of morbidity and mortality worldwide, whose complexities can lead to the difficulty of treatment (1,2). The discovery of numerous long non-coding RNA (lncRNA) transcripts in human has dramatically altered our understanding of cancer (3). LncRNAs play pivotal roles in mediating the crosstalk between various cellular components, including proteins, RNAs and lipids, which are involved in cancerous processes (4). In order to facilitate the studies of lncRNA-cancer associations, the first version of the Lnc2Cancer database (Lnc2Cancer 1.0) was reported to allow users to search all known experimentally supported lncRNAs associated with various human cancers (5).

With the increasing interests in human lncRNAs and the availability of high-throughput technologies, the number of cancer-lncRNA associations has increased rapidly (6–8). In addition, regulatory mechanisms such as genetic variant, microRNA (miRNA) interaction, transcription factor (TF) binding and methylation modification of lncRNAs in cancers have also been widely studied (9–11). Therefore, it is urgent to update Lnc2Cancer with more resources and improved tools. More importantly, some novel research directions have emerged in cancer-related lncRNA field. In recent years, increasing evidences suggested that lncRNAs could serve as potential non-invasive diagnostic (12), drug resistance (13) and prognostic-related biomarkers (14) in various cancers. For example, the potential use of circulating lncRNAs in serum, plasma and other body fluids as biomarkers for cancer (circulating lncRNA) have been investigated by several studies (15,16). Aberrant expressions of lncRNAs were reported to be responsible for drug resistance in human cancer (drug-resistant lncRNA) (17). Many lncRNAs were also reported to hold prognostic value for survival prediction of cancer patients (prognostic-related lncRNA) (18). However, there is no specialized resource devoted to collecting, storing and distributing these data. Some existing resources only collected basic annotation and functional information on lncRNA, such as LNCipedia (19), LncRNADisease (20), LNCediting (21) and lncRNAdb (22). Besides, some other studies focused on expression and genomic characterization for lncRNAs across cancers (23,24). However, a global and high-quality database focused on lncRNAs as cancer biomarkers is still lacking.

**Figure 1.**
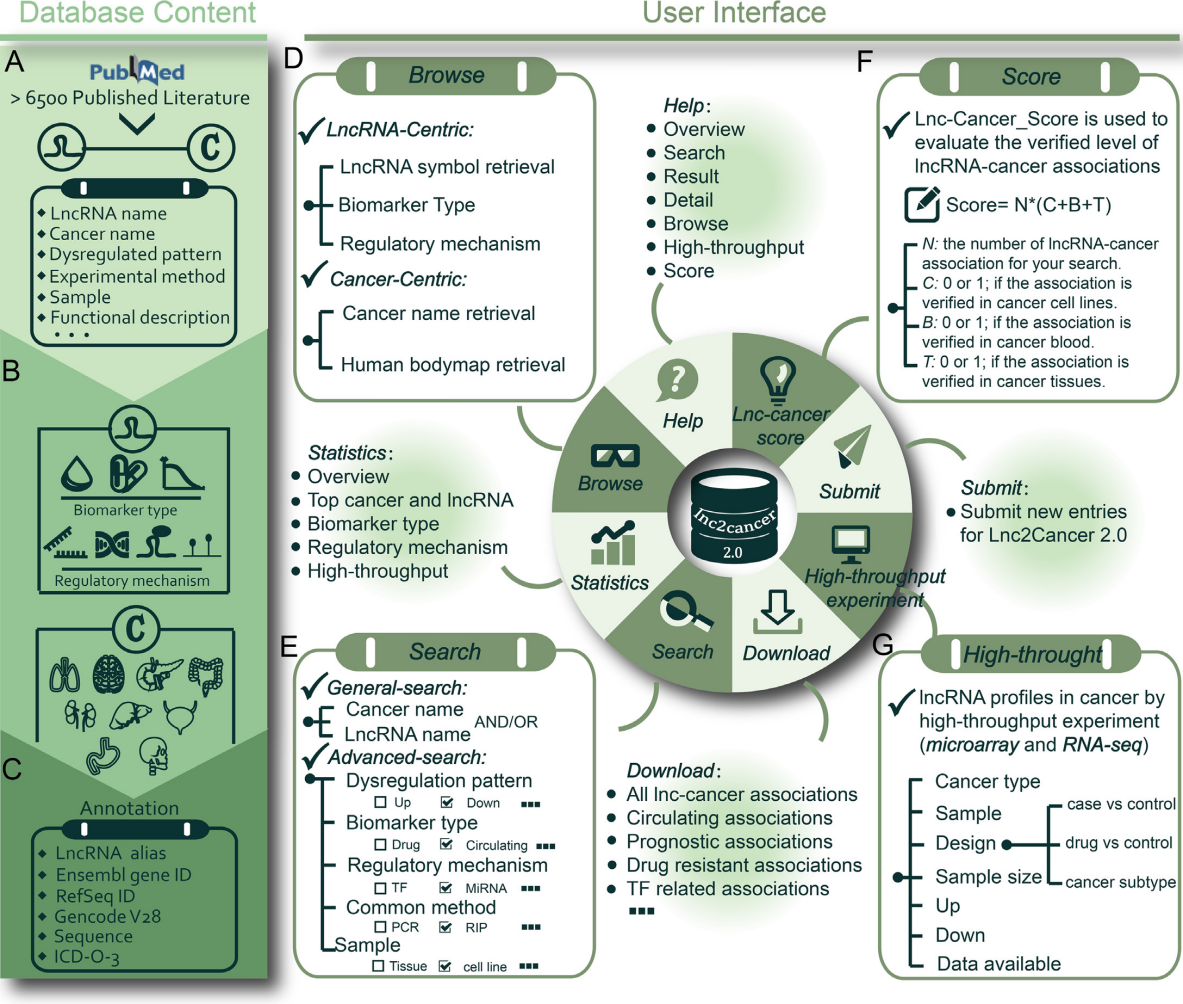
Content and interface of Lnc2Cancer 2.0. (**A**) Manually curated lncRNA-cancer associations in Lnc2Cancer 2.0. (**B**) Perfection of lncRNA-cancer associations. (**C**) Annotation of lncRNA and cancer in Lnc2cancer 2.0. (**D**) Browse for all lncRNA-cancer associations. (**E**) Search for all lncRNA-cancer associations. (**F**) LncRNA-Cancer score for lncRNA-cancer associations. (**G**) High-throughput experiments of lncRNA profiles in cancers.

To meet these needs, we updated Lnc2Cancer 1.0 to version 2.0 (Lnc2Cancer 2.0) (Figure 1 and Table 1). Lnc2Cancer 2.0 documents 4989 entries of associations between 1614 human lncRNAs and 165 human cancer subtypes by reviewing >6500 published papers. For the first time, experimentally supported circulating, drug-resistant and prognostic-related lncRNAs in human cancers are included in Lnc2Cancer 2.0. LncRNAs regulated by miRNA, TF, variant and methylation are also shown in our updated database. Furthermore, Lnc2Cancer 2.0 also contains high-throughput experiments of lncRNAs in cancers. LncRNA-Cancer_score was developed to evaluate the associations between lncRNA and cancer. We hope that Lnc2Cancer 2.0 can serve as an important resource for future researches about lncRNA and human cancer.

**Table 1.** Expanded data in Lnc2Cancer 2.0 compared with Lnc2Cancer 1.0

Features	Lnc2Cancer 1.0	Lnc2Cancer 2.0	Fold increase
LncRNA-cancer associations	1057	4989	4.72
LncRNAs	531	1613	3.04
Cancer subtypes	86	165	1.92
Circulating lncRNAs in cancer	–	366	New
Drug resistant lncRNAs in cancer	–	593	New
Prognostic-related lncRNAs in cancer	–	1928	New
LncRNAs regulated by variant	–	211	New
LncRNAs regulated by miRNA	–	1139	New
LncRNAs regulated by TF	–	225	New
LncRNAs regulated by methylation	–	319	New
High-throughput experiments	–	77	New

## IMPROVED EXPANSION AND NEW FEATURES

### Data expansion and pre-processing

Lnc2Cancer 2.0 is updated to include the increased associations between lncRNAs and cancer subtypes (Table 1). Firstly, we screened approximate 4600 studies within the PubMed database (25) (mainly from 2015 to 2018) following the similar keyword combinations as Lnc2Cancer 1.0. In addition, we also re-screened >2000 studies in the PubMed database (mainly before 2015) which had been included in Lnc2Cancer 1.0 to obtain more information and details.

Secondly, we extracted experimentally supported lncRNA-cancer associations which were supported by strong experimental evidence including RNAi, in vitro knockdown, western blot, qRT-PCR and luciferase reporter assay. If the lncRNA has been verified to be a circulating, drug-resistant or prognostic-related biomarker in cancer, we would extract this information. Similarly, the information would also be recorded when the regulatory mechanisms of miRNA, variant, TF and methylation to the lncRNAs in cancers were confirmed. In this step, we recorded detail information including lncRNAs and cancer names, sequence and positional information of the lncRNAs, experimental techniques (e.g. microarray, northern blot, qRT-PCR), experimental samples (cell line, blood and/or tissue), expression patterns (up-regulated, down-regulated or differential expressed), information from PubMed database (PubMed ID, year of publication and title of paper) and a brief functional description about associations between lncRNA and cancer from the original studies. Moreover, some high-quality high-throughput experiments (microarray and next-generation sequencing) of lncRNAs in cancers were extracted, which include the cancer versus normal samples, different cancer subtypes and cancer with drug treatment.

Thirdly, we collected other names of lncRNAs including aliases, synonyms, gene IDs, names from HGNC (26), Ensembl ID (27), GENCODE name (28), Genbank ID (29) and Refseq ID (30). We used these names to combine the synonyms for lncRNAs and ensure that same lncRNA had coincident information. We also updated the location of lncRNAs into GENCODE version 28. Then a standardized classification scheme, the International Classification of Diseases for Oncology, 3rd Edition (ICD-O-3) was used to annotate each cancer type. After data expansion and pre-processing, >6500 published papers were systematically reviewed. The current version of Lnc2Cancer includes 4989 entries of associations between 1614 human lncRNAs and 165 human cancer subtypes.

### Experimentally supported cancer biomarkers and regulatory mechanisms for lncRNAs

To provide a comprehensive resource for associations between lncRNA and cancer, we manually curated lncRNAs which can serve as cancer biomarkers. We collected three kinds of cancer biomarkers including circulating, drug-resistant and prognostic-related lncRNAs in cancer. For circulating-related lncRNAs, only the expression of lncRNAs which can be detected in blood, plasma, serum or the researchers defined circulating lncRNAs were extracted. For drug-resistant related lncRNAs (or drug-sensitive related lncRNAs), drug names were recorded. For prognostic-related lncRNAs, the lncRNAs which were verified to have a clear relationship with survival were collected. Eventually, lnc2Cancer 2.0 contains 366 circulating, 593 drug-resistant and 1928 prognostic-related lncRNA-cancer associations that have been experimentally supported.

The regulatory mechanisms of lncRNAs in cancer are complex and four main types of lncRNAs which are regulated by miRNA, variant, TF and methylation were collected. For lncRNAs regulated by miRNA and TF, we only collected the relations verified by high-quality experiments. For lncRNAs regulated by variant, if the somatic variant or genetic variant are located on or near lncRNAs and they influence the expression or structure of lncRNAs, this entry would be collected. For lncRNAs regulated by methylation, we adopted similar criterion with variant. Finally, 1139, 211, 225 and 319 entries about miRNA, variant, TF and methylation are included in Lnc2Cancer 2.0.

### High-throughput experiments of lncRNAs in cancer

In recent years, high-throughput microarray and sequencing data are producing at an unprecedented rate in cancer genomes. There is a strong need to collect high-quality lncRNA profiles in cancers based on high-throughput experiments, and this will help to explore the function and mechanism of lncRNAs in cancer at the whole genome range. Lnc2Cancer 2.0 contains 77 high-throughput experiments across 38 cancer subtypes, including cancer versus normal samples, different cancer subtypes and cancer with drug treatment.

### LncRNA-cancer score for filtering the interested associations

In Lnc2Cancer 2.0, all lncRNA-cancer associations were verified by strong experiments. To evaluate the verified levels and research hotspots, we developed a LncRNA-cancer score system. The lncRNA-cancer score is based on number of publications to verify the association and sample type (tissue, cell line or blood) used for experiment. For each lncRNA-cancer association, we calculated the confidence score as follows:

\begin{equation*}{\rm{Score}} = N*(C + B + T)\end{equation*}

where *N* is the number of studies which verified the specific lncRNA-cancer association, *C, B, T* are if the lncRNA-cancer association had been verified in cancer cell lines, blood or tissues, respectively (if association had been verified in cancer cell lines, blood or tissues, we set *C, B, T* to 1). Researchers could use the score to filter interested lncRNA-cancer associations.

## DATABASE CONSTRUCTION AND IMPROVED USER INTERFACE

All data in Lnc2Cancer 2.0 were stored and managed using MySQL (version 5.7.18). The web interfaces were built in JSP on Linux and Apache platform. The Lnc2Cancer 2.0 is freely available at http://www.bio-bigdata.net/lnc2cancer and http://www.bio-bigdata.com/lnc2cancer. The old version Lnc2Cancer 1.0 is still in service. Users can enter it from the Lnc2Cancer 2.0 homepage or go directly to http://www.bio-bigdata.net/lnc2cancer1.0/.

**Figure 2.**
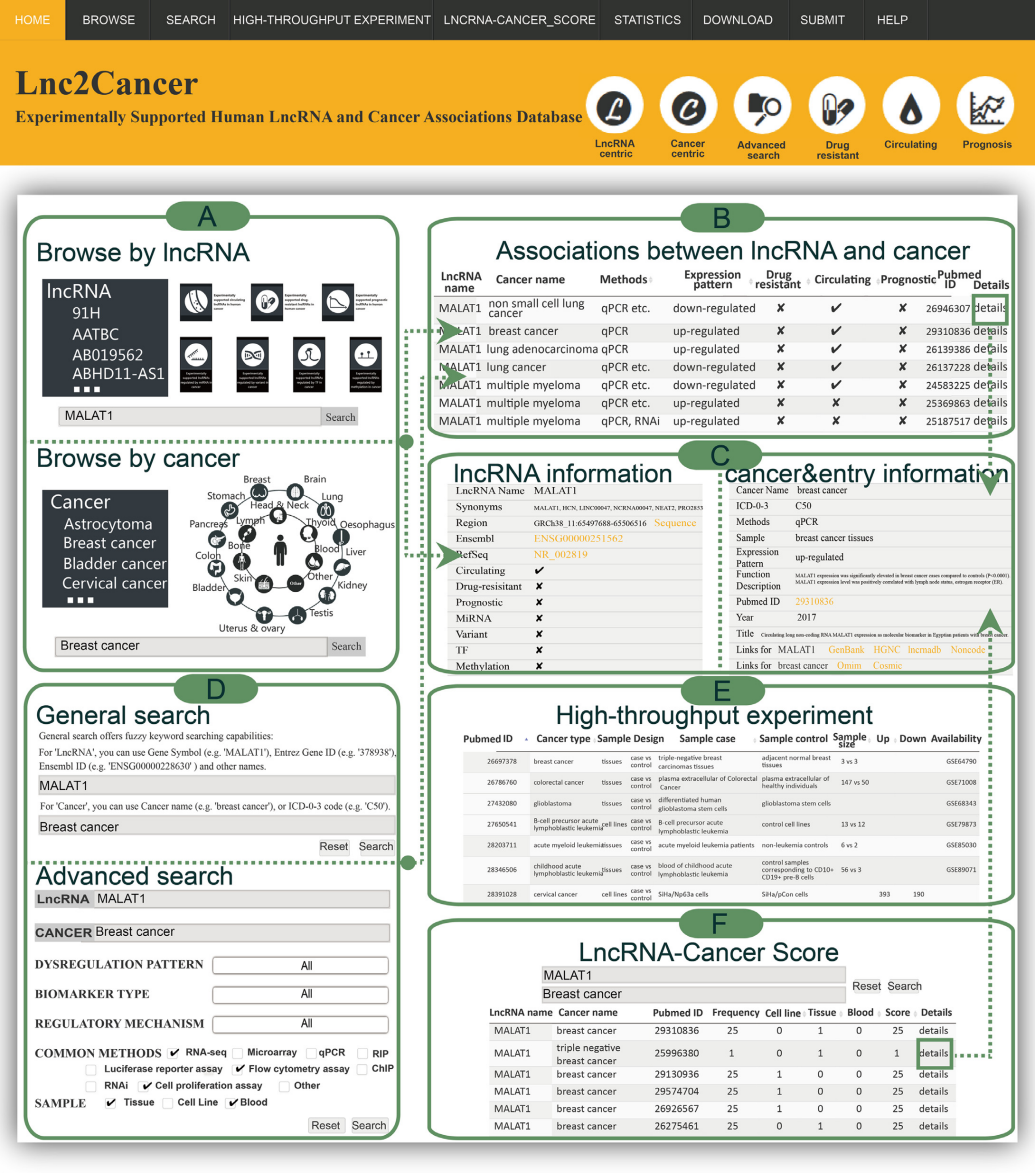
A schematic workflow of Lnc2Cancer 2.0. (**A**) The interface of the browse module, with MALAT1 input or selected as the retrieved lncRNA in ‘LncRNA-centric’ page and breast cancer as the retrieved cancer in ‘Cancer-centric’ page. (**B**) Search result page of MALAT1. (**C**) Search result page with detailed information. (**D**) The interface of the general search and advanced search module, with MALAT1 and breast cancer as examples. (**E**) The interface of high-throughput experiment module. (**F**) The interface of lncRNA-cancer score module, with MALAT1 and breast cancer as examples.

We provided a user-friendly web interface (Figure 1) that can enable users to query the database for a few steps. (i) From the ‘Browse’ page, users can browse all experimentally supported associations by ‘LncRNA-Centric’ and ‘Cancer-Centric’ (Figure 2A–C). In LncRNA-Centric page, users can browse by diverse biomarker types and regulatory mechanisms for lncRNAs. In Cancer-Centric page, there are three ways including anatomical classification in human bodymap, cancer name list and input cancer name to search and browse the data. (ii) The ‘Search’ page provides ‘general search’ and ‘advanced search’ (Figure 2D). In general search page, users can search by lncRNA name and cancer name. In advanced search, users can get more detailed and systemic search by restricting to interested descriptions containing dysregulated expression pattern, sample, common method, biomarker type and regulatory mechanism. (iii) From ‘High-throughput experiment’ page, users can browse high-throughput experiments for lncRNA profiles in cancers and check the available data by external links (Figure 2E). (iv) In ‘LncRNA-Cancer_Score’ page, users can search and filter interested associations by LncRNA-Cancer score. (v) ‘Statistics’ page provides more information about lncRNA-cancer associations. (vi) Lnc2Cancer 2.0 is a totally open resource, and users can obtain all data from the ‘Download’ page. (vii) The ‘Submit’ page enables researchers to submit novel experimentally supported lncRNA-cancer associations. (viii) In ‘Help’ page, users can get a detailed tutorial about how to use Lnc2Cancer 2.0.

## CONCLUSIONS AND FUTURE EXTENSIONS

With the increase of experimentally supported lncRNA-cancer associations, Lnc2Cancer database was updated and improved with the latest data and new features. Lnc2Cancer 2.0 provides a comprehensive resource for associations between lncRNA and cancer. As the studies regarding to lncRNA and cancer accumulate rapidly, lncRNAs in cancers are being identified and characterized at a rapid pace, and we believe that more roles of lncRNAs in cancers will be revealed in the future. We will continue to update and improve the database to keep pace of the researches. Lnc2Cancer will serve as a valuable resource for researchers interested in determining the role of lncRNAs in human cancers.

## References

[1] Ferlay J., Soerjomataram I., Dikshit R., Eser S., Mathers C., Rebelo M., Parkin D.M., Forman D., Bray F. Cancer incidence and mortality worldwide: sources, methods and major patterns in GLOBOCAN 2012. Int. J. Cancer. 2015; 136:E359–E386. 25220842 10.1002/ijc.29210

[2] Hanahan D., Weinberg R.A. Hallmarks of cancer: the next generation. Cell. 2011; 144:646–674. 21376230 10.1016/j.cell.2011.02.013

[3] Prensner J.R., Chinnaiyan A.M. The emergence of lncRNAs in cancer biology. Cancer Discov. 2011; 1:391–407. 22096659 10.1158/2159-8290.CD-11-0209 PMC3215093

[4] Huarte M. The emerging role of lncRNAs in cancer. Nat. Med. 2015; 21:1253–1261. 26540387 10.1038/nm.3981

[5] Ning S., Zhang J., Wang P., Zhi H., Wang J., Liu Y., Gao Y., Guo M., Yue M., Wang L. Lnc2Cancer: a manually curated database of experimentally supported lncRNAs associated with various human cancers. Nucleic Acids Res. 2016; 44:D980–D985. 26481356 10.1093/nar/gkv1094 PMC4702799

[6] Ashouri A., Sayin V.I., Van den Eynden J., Singh S.X., Papagiannakopoulos T., Larsson E. Pan-cancer transcriptomic analysis associates long non-coding RNAs with key mutational driver events. Nat. Commun. 2016; 7:13197. 28959951 10.1038/ncomms13197 PMC5093340

[7] Liu N., Dai Q., Zheng G., He C., Parisien M., Pan T. N(6)-methyladenosine-dependent RNA structural switches regulate RNA-protein interactions. Nature. 2015; 518:560–564. 25719671 10.1038/nature14234 PMC4355918

[8] Iyer M.K., Niknafs Y.S., Malik R., Singhal U., Sahu A., Hosono Y., Barrette T.R., Prensner J.R., Evans J.R., Zhao S. The landscape of long noncoding RNAs in the human transcriptome. Nat. Genet. 2015; 47:199–208. 25599403 10.1038/ng.3192 PMC4417758

[9] Rad S.M., Mohammadi-Sangcheshmeh A., Bamdad T., Langroudi L., Atashi A., Lotfinia M., Arefian E., Gastal E.L., Soleimani M. Pluripotency Crossroads: Junction of transcription factors, epigenetic mechanisms, MicroRNAs, and long Non-coding RNAs. Curr. Stem Cell Res. Ther. 2017; 12:300–311. 25986623 10.2174/1574888X12666170216155850

[10] Wang P., Ning S., Zhang Y., Li R., Ye J., Zhao Z., Zhi H., Wang T., Guo Z., Li X. Identification of lncRNA-associated competing triplets reveals global patterns and prognostic markers for cancer. Nucleic Acids Res. 2015; 43:3478–3489. 25800746 10.1093/nar/gkv233 PMC4402541

[11] Popadin K., Gutierrez-Arcelus M., Dermitzakis E.T., Antonarakis S.E. Genetic and epigenetic regulation of human lincRNA gene expression. Am. J. Hum. Genet. 2013; 93:1015–1026. 24268656 10.1016/j.ajhg.2013.10.022 PMC3852921

[12] Russo F., Di Bella S., Vannini F., Berti G., Scoyni F., Cook H.V., Santos A., Nigita G., Bonnici V., Lagana A. miRandola 2017: a curated knowledge base of non-invasive biomarkers. Nucleic Acids Res. 2018; 46:D354–D359. 29036351 10.1093/nar/gkx854 PMC5753291

[13] Bester A.C., Lee J.D., Chavez A., Lee Y.R., Nachmani D., Vora S., Victor J., Sauvageau M., Monteleone E., Rinn J.L. An integrated genome-wide CRISPRa approach to functionalize lncRNAs in drug resistance. Cell. 2018; 173:649–664. 29677511 10.1016/j.cell.2018.03.052 PMC6061940

[14] Lin C., Yang L. Long noncoding RNA in Cancer: Wiring signaling circuitry. Trends Cell Biol. 2018; 28:287–301. 29274663 10.1016/j.tcb.2017.11.008 PMC5869122

[15] Li S., Li Y., Chen B., Zhao J., Yu S., Tang Y., Zheng Q., Wang P., He X., Huang S. exoRBase: a database of circRNA, lncRNA and mRNA in human blood exosomes. Nucleic Acids Res. 2018; 46:D106–D112. 30053265 10.1093/nar/gkx891 PMC5753357

[16] Qi P., Zhou X.Y., Du X. Circulating long non-coding RNAs in cancer: current status and future perspectives. Mol. Cancer. 2016; 15:39. 27189224 10.1186/s12943-016-0524-4 PMC4869386

[17] Majidinia M., Yousefi B. Long non-coding RNAs in cancer drug resistance development. DNA Repair (Amst.). 2016; 45:25–33. 27427176 10.1016/j.dnarep.2016.06.003

[18] Yarmishyn A.A., Kurochkin I.V. Long noncoding RNAs: a potential novel class of cancer biomarkers. Front. Genet. 2015; 6:145. 25954300 10.3389/fgene.2015.00145 PMC4407501

[19] Volders P.J., Verheggen K., Menschaert G., Vandepoele K., Martens L., Vandesompele J., Mestdagh P. An update on LNCipedia: a database for annotated human lncRNA sequences. Nucleic Acids Res. 2015; 43:4363–4364. 25829178 10.1093/nar/gkv295 PMC4417186

[20] Chen G., Wang Z., Wang D., Qiu C., Liu M., Chen X., Zhang Q., Yan G., Cui Q. LncRNADisease: a database for long-non-coding RNA-associated diseases. Nucleic Acids Res. 2013; 41:D983–D986. 23175614 10.1093/nar/gks1099 PMC3531173

[21] Gong J., Liu C., Liu W., Xiang Y., Diao L., Guo A.Y., Han L. LNCediting: a database for functional effects of RNA editing in lncRNAs. Nucleic Acids Res. 2017; 45:D79–D84. 27651464 10.1093/nar/gkw835 PMC5210611

[22] Quek X.C., Thomson D.W., Maag J.L., Bartonicek N., Signal B., Clark M.B., Gloss B.S., Dinger M.E. lncRNAdb v2.0: expanding the reference database for functional long noncoding RNAs. Nucleic Acids Res. 2015; 43:D168–D173. 25332394 10.1093/nar/gku988 PMC4384040

[23] Yan X., Hu Z., Feng Y., Hu X., Yuan J., Zhao S.D., Zhang Y., Yang L., Shan W., He Q. Comprehensive genomic characterization of long Non-coding RNAs across human cancers. Cancer Cell. 2015; 28:529–540. 26461095 10.1016/j.ccell.2015.09.006 PMC4777353

[24] Li J., Han L., Roebuck P., Diao L., Liu L., Yuan Y., Weinstein J.N., Liang H. TANRIC: An interactive open platform to explore the function of lncRNAs in cancer. Cancer Res. 2015; 75:3728–3737. 26208906 10.1158/0008-5472.CAN-15-0273 PMC4573884

[25] Database resources of the National Center for Biotechnology Information. Nucleic Acids Res. 2018; 46:D8–D13. 29140470 10.1093/nar/gkx1095 PMC5753372

[26] Gray K.A., Yates B., Seal R.L., Wright M.W., Bruford E.A. Genenames.org: the HGNC resources in 2015. Nucleic Acids Res. 2015; 43:D1079–D1085. 25361968 10.1093/nar/gku1071 PMC4383909

[27] Zerbino D.R., Achuthan P., Akanni W., Amode M.R., Barrell D., Bhai J., Billis K., Cummins C., Gall A., Giron C.G. Ensembl 2018. Nucleic Acids Res. 2018; 46:D754–D761. 29155950 10.1093/nar/gkx1098 PMC5753206

[28] Harrow J., Frankish A., Gonzalez J.M., Tapanari E., Diekhans M., Kokocinski F., Aken B.L., Barrell D., Zadissa A., Searle S. GENCODE: the reference human genome annotation for The ENCODE Project. Genome Res. 2012; 22:1760–1774. 22955987 10.1101/gr.135350.111 PMC3431492

[29] Benson D.A., Clark K., Karsch-Mizrachi I., Lipman D.J., Ostell J., Sayers E.W. GenBank. Nucleic Acids Res. 2014; 42:D32–D37. 24217914 10.1093/nar/gkt1030 PMC3965104

[30] O’Leary N.A., Wright M.W., Brister J.R., Ciufo S., Haddad D., McVeigh R., Rajput B., Robbertse B., Smith-White B., Ako-Adjei D. Reference sequence (RefSeq) database at NCBI: current status, taxonomic expansion, and functional annotation. Nucleic Acids Res. 2016; 44:D733–D745. 26553804 10.1093/nar/gkv1189 PMC4702849

